# Unexpected renal hemorrhage after endovascular repair of complicated type B aortic dissection: two cases report

**DOI:** 10.1186/s12893-018-0440-1

**Published:** 2018-11-16

**Authors:** Hongwei Zhang, Bangsheng Jia, Ling Zeng, Zhenghua Xiao, Jiayu Shen, Hong Qian, Eryong Zhang, Jia Hu

**Affiliations:** 1 Department of Cardiovascular Surgery, West China Hospital, Sichuan University, Chengdu, Sichuan People’s Republic of China; 2 Department of Radiology, West China Hospital, Sichuan University, Chengdu, Sichuan People’s Republic of China; 3 Department of Intensive Care Unit, West China Hospital, Sichuan University, Chengdu, Sichuan People’s Republic of China

**Keywords:** Renal hemorrhage, Thoracic endovascular aortic repair, Complicated type B aortic dissection

## Abstract

**Background:**

Thoracic endovascular aortic repair (TEVAR) is the therapeutic choice for type B aortic dissection. One of the most unfavored complications of this procedure is hemorrhage, which has a low incidence but high mortality. Renal hemorrhage (RH) after endovascular aortic repair has been rarely reported. We presented two cases of unexpected RH after TEVAR for complicated type B aortic dissection, and the potential causes, diagnosis and therapeutic management were discussed.

**Case presentation:**

A 67-year-old female developed hypotension and progressively decrease of hemoglobin within 5 h after TEVAR for acute complicated type B dissection. Bedside ultrasonography and abdominal computed tomography angiography revealed a massive right perinephric hematoma. The right renal angiography detected multiple tortuous vascular branches with diffuse perinephric bleeding. The main trunk of right renal artery was embolized. The patient recovered uneventfully and presented with normal renal function 6 months later. Another patient was a 69-year-old male who was admitted for endovascular repair of a chronic complicated type B aortic dissection. The patient presented with hemodynamic instability early after TEVAR. Bedside ultrasonography showed a giant left retroperitoneal hematoma. The abdominal angiography revealed two active bleeding sits located in the distal branches of left renal artery. A super-selective embolization of the two arteries was performed, however the patient developed abdominal compartment syndrome and died of multiple organ failure.

**Conclusions:**

Unexpected RH after endovascular repair of aortic dissection might be associated with iatrogenic and idiopathic factors. Close surveillance and clinician’s awareness of this rare complication is crucial for accurate and prompt diagnosis. Renal angiography and subsequent selective embolization of bleeding vessels are effective interventions for treating this fatal condition.

**Electronic supplementary material:**

The online version of this article (10.1186/s12893-018-0440-1) contains supplementary material, which is available to authorized users.

## Background

Thoracic endovascular aortic repair (TEVAR) is the therapeutic choice for type B aortic dissection [1–3]. One of the most unfavored complications of this procedure is hemorrhage, which has a low incidence but high mortality [1–3]. To our best knowledge, renal hemorrhage (RH) after endovascular aortic repair has been rarely reported. In the present study, we presented two cases of unexpected RH after TEVAR for complicated type B aortic dissection, and the potential causes, diagnosis and therapeutic management were discussed.

## Case presentation

### Case 1

**Fig. 1:**
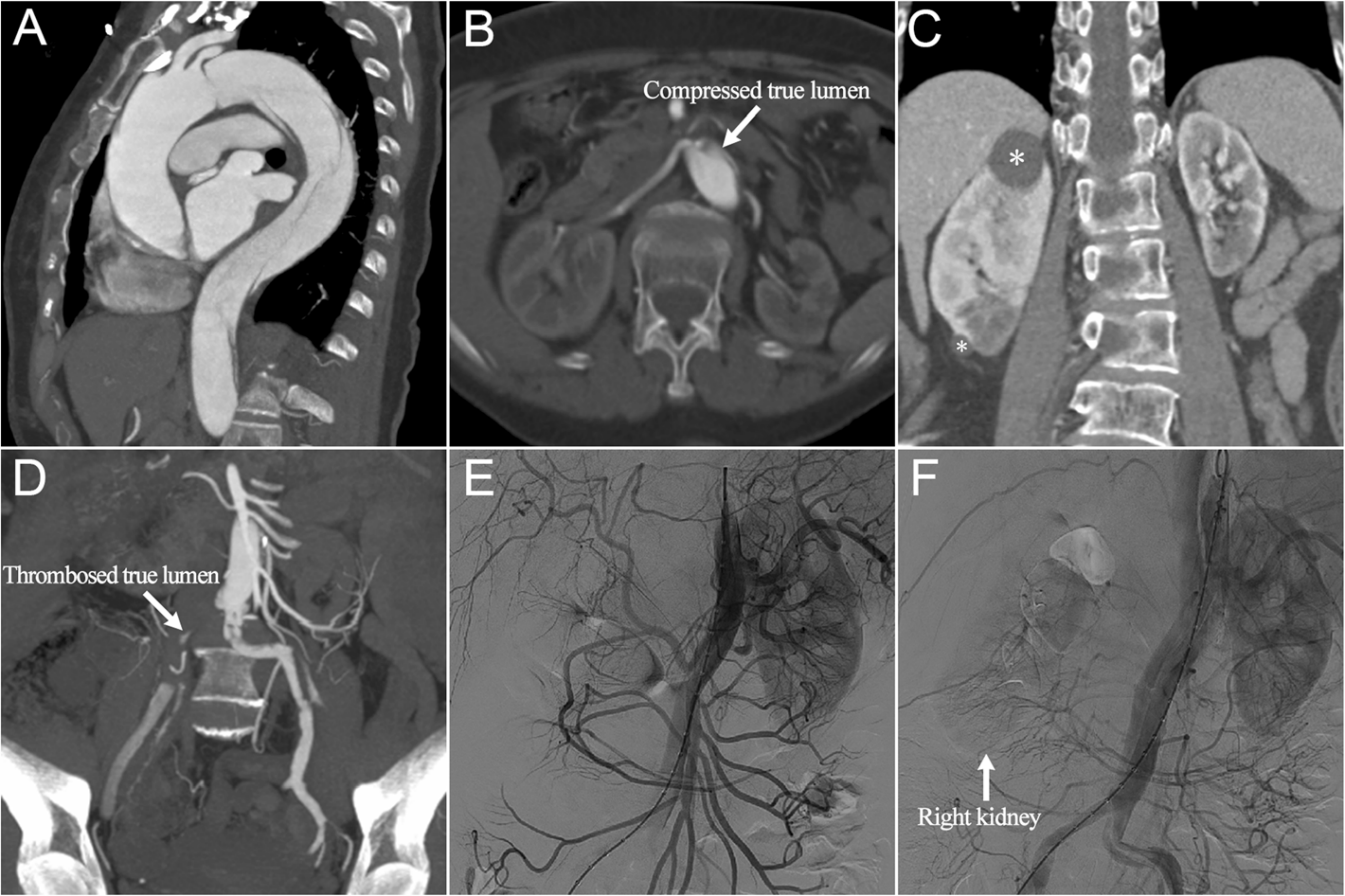
(**a**) Preoperative sagittal computed tomography angiography showed type B aortic dissection. (**b**) Left renal artery originated from a severely stenotic true lumen, right renal artery with dynamic occlusion was supplied via a false lumen. (**c**) Two cysts (asterisks) were separately located at the upper and inferior poles of right kidney. (**d**) Incomplete thrombosis was detected in the bilateral common iliac arteries. (**e**) Abdominal angiography showed poor perfusion of the right kidney and bilateral iliac arteries, and (**f**) completion angiography demonstrated the reopening of the distal true lumen and an improved flow in right renal artery and bilateral iliac arteries

A 67-year-old female patient presented in the emergency department with persistent chest pain for 12 h, followed by aggravating lower limbs numbness and oliguria, with a urine output of 40 ml after onset. Computed tomography angiography (CTA) revealed an acute type B aortic dissection with a primary entry tear approximating to the left subclavian artery (LSA) and extending to the iliac arteries (Fig. 1a). Left renal artery originated from a severely stenotic true lumen, right renal artery with dynamic occlusion was supplied via a false lumen (Fig. 1b), and there were two cysts (28 mm × 25 mm, 10 mm × 10 mm) separately located at the upper and inferior poles of right kidney (Fig. 1c). Incomplete thrombosis was detected in the bilateral common iliac arteries (Fig. 1d).

The patient underwent emergent TEVAR 3 h after admission because of the malperfusion symptoms of right kidney and lower extremity. A hydrophilic angled guidewire (0.035 in. × 180 cm; Radifocus, Terumo) was inserted into the aortic true lumen via the right femoral artery, and the angiography showed poor perfusion of the right kidney and bilateral iliac arteries (Fig. 1e). The distal restrictive covered stent (straight 24 mm × 80 mm; Endurant, Medtronic) was introduced and deployed at the proximal descending aorta. Subsequently, the thoracic stent graft (straight 36 mm × 200 mm; Valiant Captiva, Medtronic) was introduced, overlapped 30 mm with the restrictive stent and deployed at the distal aortic arch (Additional file 1). The LSA was sacrificed because of inadequate proximal landing zone and the dominant right vertebral artery. Completion angiography demonstrated a satisfactory coverage of the primary entry tear, and the reopening of the distal true lumen and an improved flow in right renal artery and bilateral iliac arteries (Fig. 1f).

**Fig. 2:**
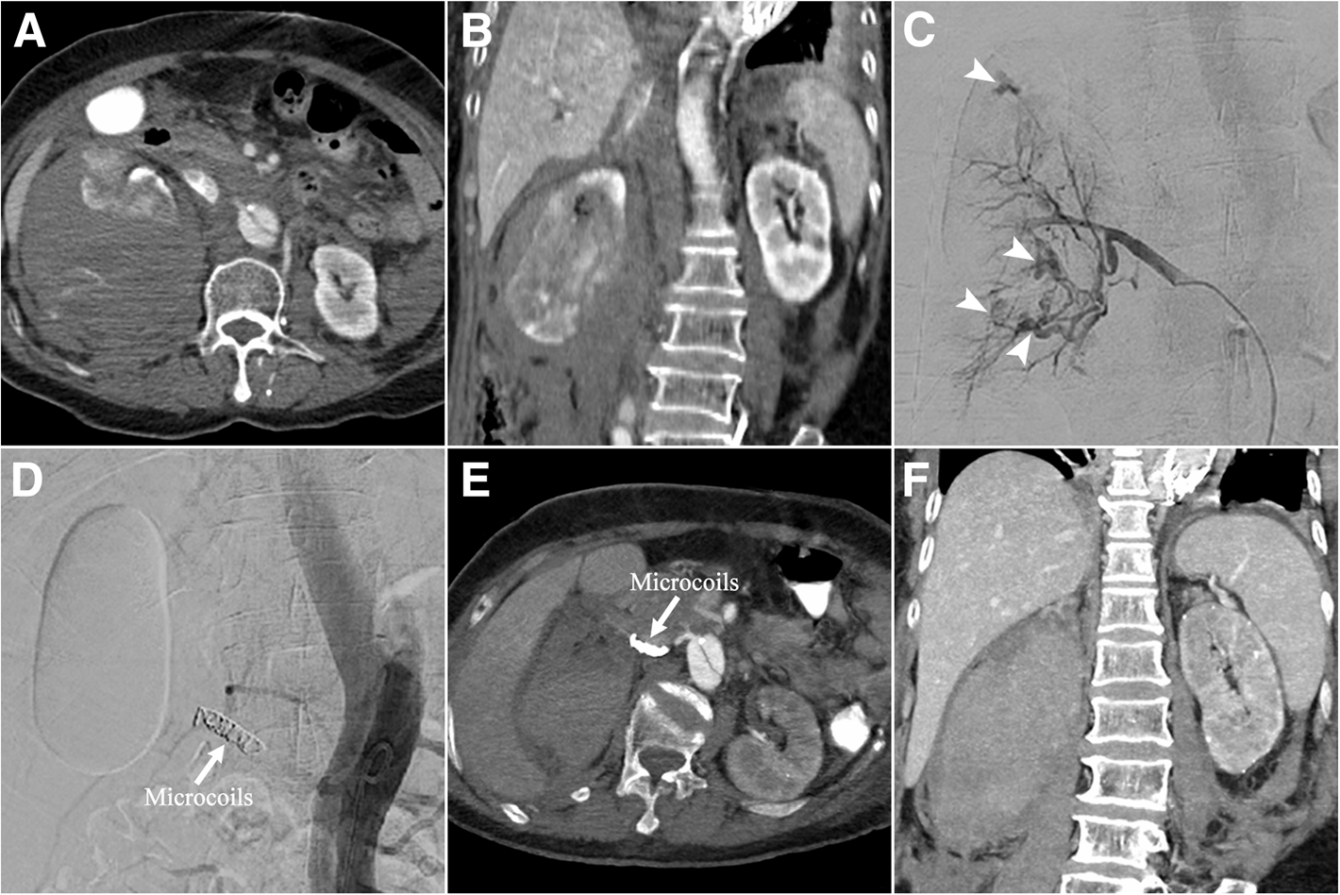
(**a**) Transverse and (**b**) coronal computed tomography angiography showed a massive right perinephric hematoma. (**c**) Right renal angiography detected multiple tortuous vascular branches with diffuse perinephric bleeding (arrowheads), and (**d**) the main trunk of right renal artery was embolized with microcoils. (**e**, **f**) Pre-discharge computed tomography angiography showed no signs of hemorrhage of the right kidney

Although the distal malperfusion syndrome was successfully treated, the patient showed hemodynamic instability and progressively decrease of hemoglobin from 118 to 82 g/L within 5 h after surgery. Bedside ultrasonography and abdominal CTA revealed a massive right perinephric hematoma measuring 10 cm × 15 cm (Fig. 2a, b). As her vital signs were unstable even after 6 units of blood transfusion and another 2000 ml of fluid resuscitation within 4 h, an emergency transcatheter embolization was performed. The right renal angiography detected multiple tortuous vascular branches with diffuse perinephric bleeding (Fig. 2c). The main trunk of right renal artery was embolized with metallic microcoils (0.035 in.; Cook) and histoacryl glue (B. Braun) (Fig. 2d, Additional file 1).

The patient was hemodynamically stable, and the hemoglobin returned to normal. Although a period of renal insufficiency with the maximum serum creatinine level reaching 343 μmol/L was observed after surgery, the patient maintained normal urine output (1200-1500 ml/day) without any hemodialysis. The patient recovered uneventfully, and no signs of hemorrhage of the right kidney were detected by pre-discharge CTA 7 days after TEVAR (Fig. 2e, f). Six-month follow-up showed the patient was in good condition and presented with normal renal function.

### Case 2

**Fig. 3:**
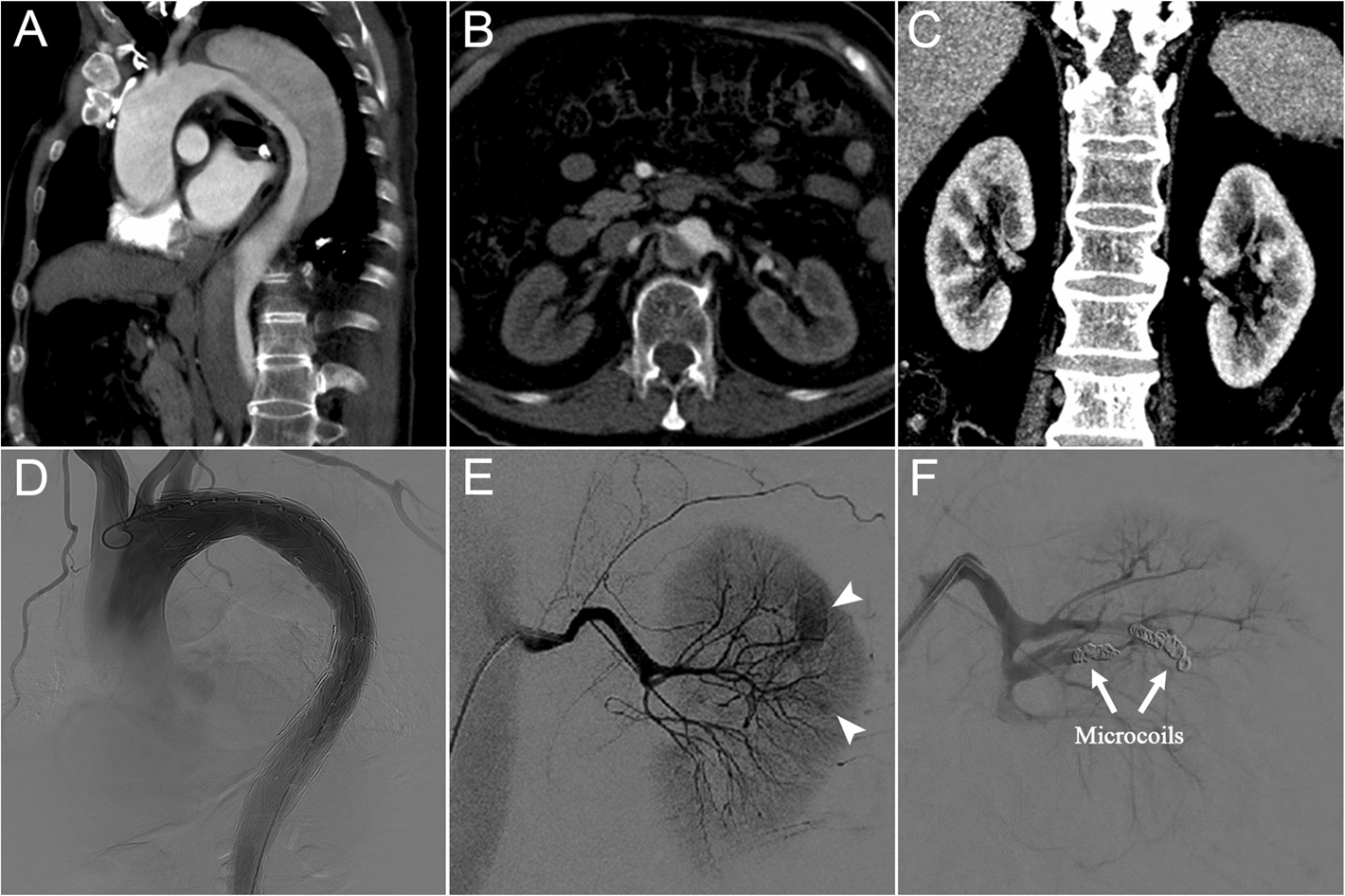
(**a**) Preoperative sagittal computed tomography angiography showed a type B dissection ranging from the distal aortic arch to the iliac arteries. (**b**) The left renal artery originated from the true lumen, and (**c**) without cysts or tumors. (**d**) Completion angiography showed the entry tear was successfully occluded. (**e**) Left renal angiography revealed two active bleeding sits located in the distal branches of left renal artery (arrowheads). (**f**) Completion angiography showed the effective occlusion of the feeding vessel and termination of the left renal bleeding

Another patient was a 69-year-old male who was admitted for endovascular repair of a chronic complicated type B aortic dissection. He had history of poorly controlled hypertension for 10 years due to irregular intake of antihypertensive medications. The dissection ranged from the distal aortic arch to the iliac arteries (Fig. 3a). The left renal artery originated from the true lumen, and the right renal artery was supplied via both the true and false lumen, no cysts or tumors were found in both kidneys (Fig. 3b, c).

TEVAR was accepted as a reasonable treatment strategy after the consent of the patient. Through right femoral artery, the first (distal) (tapered 28 mm–24 mm × 150 mm; Valiant Captiva, Medtronic) and the second (proximal) (straight 34 mm × 200 mm; Valiant Captiva, Medtronic) thoracic stent grafts were accurately deployed without sacrificing LSA, and the entry tear was successfully occluded (Fig. 3d**,** Additional file 2).

The patient complained of left flank pain and presented with hemodynamic instability early after TEVAR. His systolic blood pressure rapidly decreased to less than 80 mmHg, and the hemoglobin value sharply dropt from 122 to 64 g/L within only 2 h postoperatively, even intravenous bolus administration and massive blood transfusion could not maintain his vital signs stable. Bedside ultrasonography showed a giant left retroperitoneal hematoma. Progressively hemodynamic instability forced cessation of further radiological examination. The patient was immediately transferred to the operating room for emergency transcatheter embolization because of highly suspicious of left RH. The abdominal angiography revealed two active bleeding sits located in the distal branches of left renal artery, and no bleeding sites were found at the aorta and other branches (Fig. 3e**,** Additional file 2). A super-selective embolization of two renal arterial branches was performed with metallic microcoils (0.018 in.; Cook) and histoacryl glue (B. Braun). Completion angiography showed effective occlusion of the feeding vessel and termination of the left renal bleeding (Fig. 3f**,** Additional file 2). The patient developed abdominal compartment syndrome and died of multiple organ failure 2 days after surgery.

## Discussion

Endovascular repair has been increasingly used for treating aortic diseases [1–3]. A series of complications after TEVAR are associated with vascular injury, resulting in branch vessel rupture and active bleeding [1–3]. Active RH is a rare but life-threatening complication after endovascular procedures. Identification of its potential etiology is important to prevent the occurrence of this catastrophic event.

Unexpected hemorrhage of visceral organs after endovascular aortic repair is usually caused by iatrogenic injuries of the branch vessels during wires, catheters or sheaths insertion. [4–8]. It was reported that the incidence of iatrogenic injuries after percutaneous renal artery revascularization had been 6.5–22.8% [4, 7, 8]. As reported by several studies [4, 5, 7, 8], the iatrogenic perforation and rupture of small branch vessels are usually caused by floppy-tipped guidewires. The hydrophilic guidewires and catheters may accidently enter the renal arterial branches during wire exchanging procedures, when it was performed without any fluoroscopic navigation [4, 5, 7, 8]. Particularly in patients with aortic dissection, the true lumen is usually compressed by enlarged false lumen [3], and the guidewire may easily pass through the narrowed true lumen into the downward angled renal artery. In the present study, the most likely cause of RH in the second patient was guidewire-induced injuries of small branches of renal arteries. Therefore, careful manipulation of guidewires under full fluoroscopic navigation is crucial to avoid unexpected branch vessel injury during endovascular procedures.

Although iatrogenic artery injury was reported as the leading cause of RH, the first patient in this study showed an uncommon cause of post-TEVAR hemorrhage of kidneys. Existing evidence has demonstrated that reperfusion syndrome contributes to idiopathic bleeding following endovascular artery revascularization [9–12]. The typical RH caused by reperfusion injury occurs after stenotic renal artery stenting [9, 10]. Despite the difference between TEVAR and renal revascularization, but they share the similar pathogenesis in causing reperfusion hemorrhage. However, RH attributed to reperfusion injury after successful endovascular repair of aortic dissection was not reported previously. In aortic dissection patients with renal ischemia or abnormalities, such as tumors, cysts or aneurysms, the capsular branches are extremely fragile [3, 13]. RH may occur when these branches are suddenly subjected to the remarkable increased pressure after reopening of the distal true lumen. Owing to the multifocal bleeding of right kidney, the idiopathic hemorrhage of the first patient who presented with renal malperfusion might be ascribed to ischemia-reperfusion injury. Nevertheless, the cause of renal bleeding located at the upper pole could not exclude cyst rupture. Consequently, for the patients with complicated type B dissection, especially for those accompanied with renal malperfusion or abnormalities, more attention should be paid to the possibility of unexpected RH after TEVAR.

The survival of the patient often depends on early and accurate diagnosis, since RH is an insidious and lethal complication of TEVAR. The patients are recommended to be routinely sent to the observation room for careful surveillance at early postoperative period. RH should be alerted if the patient presents with progressively decrease of hemoglobin, hypotension, tachycardia and acute ipsilateral abdominal/flank pain [6, 14, 15]. Rapid diagnosis of RH relies on proper imaging. CTA has been regarded as an excellent diagnostic modality for hemorrhage with high sensitivity [15–18]. Charbit et.al reported that small renal hematoma less than 25 mm without active extravasation of contrast on CTA could exclude an indication for embolization [17]. Since CTA investigation is time consuming, it could delay an early effective treatment for patients with active bleeding. CTA was not used in the second patient who had already presented with hemodynamic instability, and bedside ultrasonography was performed for both patients. Ultrasonography can be fast and convenient to detect renal subcapsular fluid collection or perirenal hematoma [18, 19], thus it is useful for rapid identification of RH, especially for patients who require expeditious intervention. Catheter angiography remains the golden standard for the diagnosis of RH, and it can clearly locate the bleeding sites and provide the chance for possible embolization simultaneously [14, 15]. We suggest that a diagnostic angiography being timely undertaken for all patients with suspected RH after TEVAR, especially for those with active hemorrhage in case of treatment delay.

An optimal decision-making relies on a comprehensive clinical assessment. Most renal vascular injuries are spontaneous cured, and conservative treatment is recommended to patients who are hemodynamic stable with no evidence of ongoing hemorrhage [6, 8, 15–17]. Urgent intervention is indicated for patients with massive bleeding, uncontrolled hemorrhage lasting for more than 72 h, or progressively deterioration of the renal function [20]. Compared with open surgery, transcatheter embolization has been established as a preferred treatment for RH after TEVAR [6, 14–17, 20–22]. Super-selective renal embolization is a minimally invasive and effective procedure for RH, and definitely minimizes the damage of normal renal parenchyma, thus maintaining renal functional and preventing post-embolization syndrome [14–16, 20, 22]. Emergent embolization of renal arteries was a life-saving treatment of choice for our cases.

## Conclusion

Unexpected RH after endovascular repair of aortic dissection might be associated with iatrogenic and idiopathic factors. Close surveillance and clinician’s awareness of this rare complication is crucial for accurate and prompt diagnosis. Renal angiography and subsequent selective embolization of bleeding vessels are effective interventions for treating this fatal condition.

## Additional files

Additional file 1: The procedures of thoracic endovascular aortic repair and right renal embolization for the first patient. (MP4 23804 kb)

Additional file 2: The procedures of thoracic endovascular aortic repair and left renal embolization for the second patient. (MP4 11521 kb)

## Abbreviations

CTA: Computed tomography angiography
LSA: left subclavian artery
RH: Renal hemorrhage
TEVAR: Thoracic endovascular aortic repair
